# Determinants and mediating mechanisms of quality of life and disease-specific symptoms among thyroid cancer patients: the design of the WaTCh study

**DOI:** 10.1186/s13044-023-00165-5

**Published:** 2023-07-10

**Authors:** Floortje Mols, Dounya Schoormans, Romana Netea-Maier, Olga Husson, Sandra Beijer, Katrijn Van Deun, Wouter Zandee, Marleen Kars, Pleun C. M. Wouters van Poppel, Suat Simsek, Patrick van Battum, Jérôme M. H. Kisters, Jan Paul de Boer, Elske Massolt, Rachel van Leeuwaarde, Wilma Oranje, Sean Roerink, Mechteld Vermeulen, Lonneke van de Poll-Franse

**Affiliations:** 1 CoRPS - Center of Research On Psychological Disorders and Somatic Diseases, Department of Medical and Clinical Psychology, Tilburg University, Tilburg, the Netherlands; 2 Netherlands Comprehensive Cancer Organisation (IKNL), Utrecht, the Netherlands; 3 Department of Internal Medicine, Radboud University Medical Center, Nijmegen, The Netherlands; 4 Department of Psychosocial Research and Epidemiology, Netherlands Cancer Institute, Amsterdam, the Netherlands; 5 Department of Medical Oncology, The Netherlands Cancer Institute, Amsterdam, The Netherlands; 6 Department of Surgical Oncology, Erasmus Medical Center, Rotterdam, the Netherlands; 7 Maastricht University Medical Center (MUMC), Maastricht, the Netherlands; 8 Department of Methodology and Statistics, Tilburg University, Tilburg, The Netherlands; 9 Department of Endocrinology, Groningen University, University Medical Center Groningen, Groningen, The Netherlands; 10 Maxima Medical Center, Veldhoven, The Netherlands; 11 Noordwest Ziekenhuisgroep, Alkmaar, The Netherlands; 12 Zuyderland MC Hospital, Heerlen, The Netherlands; 13 Catharina Hospital, Eindhoven, The Netherlands; 14 Antoni Van Leeuwenhoek Hospital, Netherlands Cancer Institute, Amsterdam, The Netherlands; 15 Albert Schweitzer Hospital, Dordrecht, The Netherlands; 16 Department of Endocrine Oncology, University Medical Center Utrecht, Utrecht, The Netherlands; 17 ETZ, Tilburg, The Netherlands; 18 Rijnstate, Arnhem, The Netherlands; 19 CWZ, Nijmegen, The Netherlands

**Keywords:** Activity trackers, BIA weighing scales, Food diaries, Inflammation, Kynurenine pathway, Patient reported outcomes, PROFILES registry, Thyroid cancer

## Abstract

**Background:**

Thyroid cancer (TC) patients are understudied but appear to be at risk for poor physical and psychosocial outcomes. Knowledge of the course and determinants of these deteriorated outcomes is lacking. Furthermore, little is known about mediating biological mechanisms.

**Objectives:**

The WaTCh-study aims to;

1. Examine the course of physical and psychosocial outcomes.
2. Examine the association of demographic, environmental, clinical, physiological, and personality characteristics to those outcomes. In other words, *who* is at risk?
3. Reveal the association of mediating biological mechanisms (inflammation, kynurenine pathway) with poor physical and psychological outcomes. In other words, *why* is a person at risk?

**Design and methods:**

Newly diagnosed TC patients from 13 Dutch hospitals will be invited. Data collection will take place before treatment, and at 6, 12 and 24 months after diagnosis. Sociodemographic and clinical information is available from the Netherlands Cancer Registry. Patients fill-out validated questionnaires at each time-point to assess quality of life, TC-specific symptoms, physical activity, anxiety, depression, health care use, and employment. Patients are asked to donate blood three times to assess inflammation and kynurenine pathway. Optionally, at each occasion, patients can use a weighing scale with bioelectrical impedance analysis (BIA) system to assess body composition; can register food intake using an online food diary; and can wear an activity tracker to assess physical activity and sleep duration/quality. Representative Dutch normative data on the studied physical and psychosocial outcomes is already available.

**Impact:**

WaTCh will reveal the course of physical and psychosocial outcomes among TC patients over time and answers the question *who* is at risk for poor outcomes, and *why.* This knowledge can be used to provide personalized information, to improve screening, to develop and provide tailored treatment strategies and supportive care, to optimize outcomes, and ultimately increase the number of TC survivors that live in good health.

## Background

Thyroid cancer (TC) is rare; the age-standardized incidence rate in Europe is 6.3 per 100.000 per year [1]. However, incidence rates are increasing [2–5]. Whereas differentiated TCs like papillary and follicular have very good prognosis with 20-year relative survival rates of 95% [6], the rarer medullary cancers have 10-year survival rates of 75–85% [7–9], while patients with rare anaplastic tumors often do not survive > 6 months [8]

Treatment of the majority of differentiated TC generally consists of surgery and, depending on tumour size and classification, followed by radioactive iodine therapy. This is accompanied by lifelong requirement of substitution therapy with levothyroxine, in selected patients with dosing regimens suppressing thyroid stimulating hormone (TSH) production [10, 11] causing (subclinical) hyperthyroidism. This can have profound effects on well-being [12–14]. For medullary or anaplastic TC, various treatment options are available that may also result in symptom relief, but little is known on its impact on patients’ lives.

Due to the good prognosis and the perceived mild treatment (that is, compared to chemotherapy for example among other malignancies), it has long been assumed that patients with differentiated TC are less distressed than other cancer patients, and that long-term quality of life (QOL) is good. Especially, impaired QOL has been reported in association with thyroxine withdrawal preceding treatment with radioactive iodide [15]. The latter is required to ensure sufficient TSH stimulation to increase the radioactive iodide uptake in thyroid (tumour) remnants. The introduction during the past decade of recombinant human TSH (rhTSH) for this indication, particularly for low-risk patients, has reduced this problem [16–19]. Nonetheless, recent studies reported much lower levels of physical and psychosocial outcomes compared to normative populations [20–22]. The vulnerable and understudied group of TC survivors might thus be in need for additional care [18–22]. Moreover, TC treatment is often based on poor quality evidence, confirmed by large treatment variation, leaving room for shared decision-making. In addition to that, most survivors require lifelong hormonal substitution therapy, which has a profound impact on physical and psychological functioning even when the TSH is kept within the normal range and needs to be adjusted individually to maintain QOL [10, 11, 13, 14, 21]. Besides the lack of knowledge on the course of physical and psychosocial outcomes over time, the role of other determinants such as diet, body weight, body composition, and physical activity, and the role of possible mediating biological mechanisms is mostly lacking for the TC survivors. We do however know that they play a role in other cancer patient populations, and they provide a lead for interventions and thus better outcomes.

To shed more light onto the outcomes of TC survivors, we set up the WaTCh-study, which stands for ‘Well-being after Thyroid Cancer’. Our main objective is to reveal the course of physical and psychosocial outcomes over time, and to answer the question *who* is at risk for low physical and psychosocial outcomes and *why.* To achieve this objective, we have formulated three complementary research directions:

*Objective 1:* To examine the course of physical and psychosocial outcomes (QOL, TC-specific symptoms (e.g., fatigue, sleep disturbances), physical activity, anxiety, depression, health care use, and employment) over time among TC patients. We hypothesize that these outcomes will worsen after diagnosis and during initial treatment, but that they will partially recover during follow-up. This is based on results observed in other cancer patient populations [23, 24]. However, given the lifelong requirement of thyroxine substitution that is known to have numerous effects on well-being in case of over- or under substitution, this could add another dimension to the findings that might be specific for the TC survivors.

*Objective 2:* To examine the role of demographic (age, sex), environmental (food intake, body weight, body composition), clinical (tumour stage, treatment), physiological (physical activity, sleep), and personality characteristics (optimism) on physical and psychosocial outcomes of TC patients. In other words, *who* is at risk? We expect demographic, clinical and personality characteristics to have a profound negative impact on patients’ physical and psychosocial outcomes [12, 25–29]. Also, we hypothesize that TC patients will report worse outcomes compared to an age- and sex matched normative population at all assessments [21, 24, 30]. However, the role of other determinants like food intake, body weight, body composition, physical activity and sleep on patients’ physical and psychosocial outcomes is less clear. Currently, no studies have investigated whether food intake is associated with physical and psychosocial outcomes in TC patients, and little is known about the influence of body composition. Some studies report weight gain under TSH suppression after total thyroidectomy [31] whereas other studies do not [32]. Furthermore, lower muscle mass due to a higher dose of thyroid hormones leads to fatigue and impaired activities and thus probably worse outcomes [33]. From studies among other cancer populations, we learned that food intake, body mass index (BMI), waist circumference, physical activity, and sleep influence health related QOL [34–42].

*Objective 3:* To reveal the association of mediating biological mechanisms (inflammation, kynurenine pathway) with poor physical and psychosocial outcomes in TC patients. In other words, *why* is a person at risk? To our knowledge, no studies have been performed on biological mechanisms involved in physical and psychosocial well-being among TC survivors. Nevertheless, evidence is mounting for a biological basis (i.e., increased levels of smouldering inflammation) related to experiences like fatigue, pain, and QOL among (non-)cancer populations [43–49]. Knowledge on underlying biological mechanisms can tailor future interventions. For example, physical activity is known to reduce pro-inflammatory cytokines related to experienced symptoms like fatigue [50]. However, the role of inflammation on physical and psychosocial outcomes is yet unknown among TC patients.

## Design and methods

### Conceptual model

**Fig. 1:**
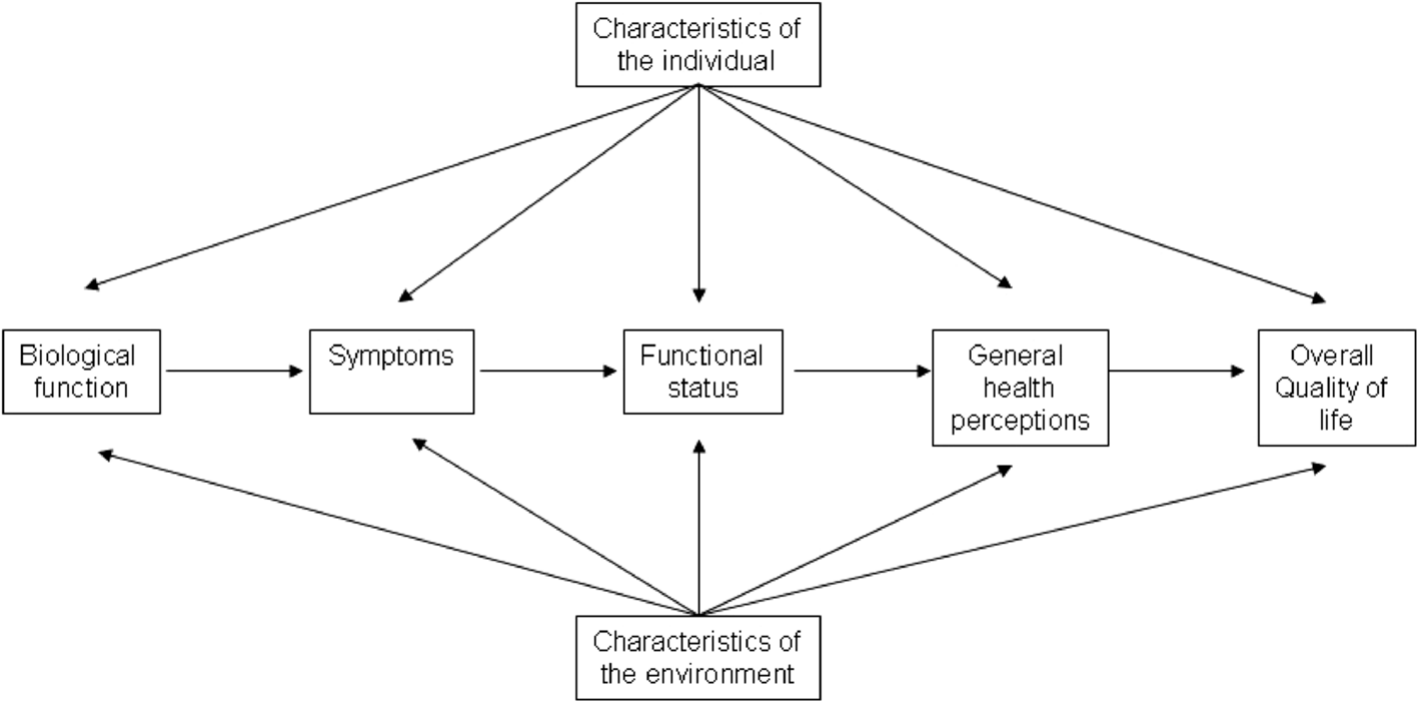
Revised Wilson & Cleary (1995) Model Adapted from Wilson, I.B., & Cleary, P.D (1995). Linking Clinical Variables with Health-Related Quality of Life: A Conceptual Model of Patient Outcomes. *JAMA. 273*, 59–65 (Ferrans, C. E., et al. (2005). Conceptual model of health-related quality of life. *J Nurs Scholarsh. 37*, 336–342.)

To understand *if*, *how* and *why* TC survivors experience declining physical and psychosocial outcomes, we use the revised Wilson&Cleary conceptual model of patient outcomes (Fig. 1 [51, 52]). Information on the selection of determinants, underlying mediating mechanisms, and outcome variables used in WaTCh are based upon this model and are described below. Next, we describe the recruitment, study population, and the various data collection methods.

### Determinants

#### Characteristics of the individual

Characteristics of the individual in the revised Wilson&Cleary model are categorized as demographic, psychological and biological factors that influence health outcomes [51]. Numerous studies among various cancer types showed that demographic (i.e., sex, age, education, and marital status) and personality characteristics (optimism) have an influence on physical and psychosocial outcomes [25–28]. Therefore, they are included as determinants.

#### Characteristics of the environment

Wilson&Cleary categorize characteristics of the environment as social or physical. Social characteristics are the interpersonal or social influences (e.g. presence of a partner) on health outcomes, whereas physical characteristics are settings such as the home, neighborhood and work place [52]. We hypothesize that environmental characteristics affect health behaviour such as food choices, smoking and alcohol use. An increasing body of evidence supports the relationship between environmental factors such as (mal)nutrition and carcinogenesis [53]. Also, obesity and metabolic syndrome are linked to an increased TC risk [54]. Inflammation is also important in cancer promotion and metastasis [55]. Foods, nutrients, and dietary patterns have potential inflammatory effects reflected by the Dietary Inflammatory Index score (DII-score). A higher DII-score has been associated with higher cancer risk and mortality [56]. No studies have investigated whether individual diets based on their inflammatory potential were associated with physical and psychosocial outcomes. In addition, little is known about the influence of body composition (i.e., percentages of fat and fat free mass) on these outcomes in TC patients. Furthermore, lower muscle mass due to a higher dose of thyroid hormones to suppress TSH production leads to fatigue, weakness, and impaired daily activities [33]. Whether the method of preparation for radioactive iodine ablation therapy and changes in dose of replacement therapy influences weight and body composition and whether this affects short-term and long-term physical and psychosocial outcomes is unknown.

### Mediating biological mechanisms

In the revised Wilson&Cleary model, biological factors are incorporated in the conceptualization of the relationships between symptoms and QOL as part of a continuum of biological factors at one end, and increasing in complexity to include physical and psychosocial outcomes at the other end [51]. Evidence is mounting for a biological basis of a range of subjective experiences including fatigue, pain, and overall QOL among non-cancer populations [43, 44].

#### Inflammation

Chronic inflammation is regarded as an "enabling" characteristic of cancer since it affects the development of cancer and promotes all stages of tumorigenesis [57, 58]. Also, inflammation plays an important role in mediating the severity of cancer-related symptoms. Inflammation is a strong mechanism underlying fatigue [44]. Fatigue, which is common among TC patients, negatively impacts other physical and psychosocial outcomes [59, 60]. Increased levels of pro-inflammatory cytokines were reported in cancer patients, and they also have effects on the central nervous system through which they modulate perception of fatigue [61]. One of the factors reducing levels of inflammation is physical activity [50].

#### Kynurenine pathway

The immune system is linked to the kynurenine pathway which consists of a series of linked chemical reactions converting tryptophan into a variety of substances called ‘kynurenines’ [62]. The kynurenine pathway plays an important role in how tumour cells evade immune systems [62]. As elevated pro-inflammatory cytokine levels are linked to symptoms often experienced by cancer survivors, the kynurenine pathway may be involved as well [63].

### Outcomes

#### Symptoms, functional status, general health perceptions and overall QOL

TC and its treatment can have a profound impact on patients’ physical and psychosocial outcomes [12]. The most important outcomes, and most often assessed among other cancer patient populations, are QOL, symptoms, fatigue, sleep, physical activity, anxiety, depression, health care utilization, and employment. These constructs are our focus.

### Study population

WaTCh is a longitudinal population-based study, in which *all* newly diagnosed adult TC patients from 13 Dutch hospitals will be invited, except those with cognitive impairment, and those not able to read or write Dutch. As medullary and anaplastic TC are very rare but understudied, they will be included, but only with the purpose to perform descriptive analyses on their well-being.

Normative data will be used to determine the functional impairment and symptom burden after TC cancer in the context of normal aging [64] and comorbidity. Each year, information on symptoms, functional decline, comorbidities and behavioral variables is collected within the CentERpanel, a longitudinal cohort of 2.000 persons without cancer, designed to be representative of the Dutch-speaking population in the Netherlands [65–67] generated by CentERdata (www.centerdata.nl). Of this group of 2000 persons, an age and sex-matched selection will be made to match our TC patients.

### Recruitment

At the hospital, newly diagnosed TC patients will be informed by a physician. Patients receive a patient information letter and informed consent form. Informed consent forms are sent back to the PROFILES (Patient Reported Outcomes Following Initial treatment and Long term Evaluation of Survivorship) registry that coordinates this study from that moment onwards [68]. On the informed consent form, patients can indicate whether they prefer paper or online questionnaires, and whether they are interested in optional assessments (see below).

### Data collection

**Fig. 2:**
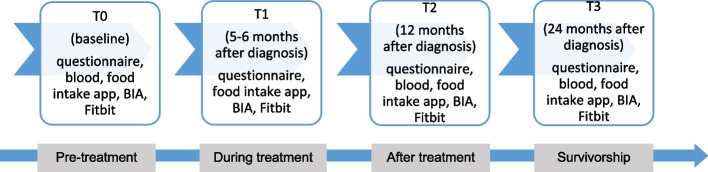
Design of the WaTCh-study

In short, patients fill-out internationally validated questionnaires on physical and psychosocial outcomes at four occasions: before treatment, and 6, 12 and 24 months after diagnosis (Fig. 2). Patients have the option to compare their questionnaire scores to their previous scores, to scores of other TC patients, and/or a normative population (see ‘Patient feedback’ below). Patients are asked to donate blood three times (before treatment, 1 and 2 years after diagnosis). Survivors’ sociodemographic and clinical information is available from the questionnaires and the Netherlands Cancer Registry (NCR). Optionally, patients can use a weighing scale with bioelectrical impedance analysis (BIA) system to assess body composition, register food intake using an online food diary, and wear an activity tracker to assess physical activity and sleep duration and quality at all four occasions. These parts are optional to minimize patient burden, to support inclusion, and prevent dropout. Details of the data collection are described below in more detail.

### Determinants

#### Characteristics of the individual

Basic sociodemographic and clinical information is either self-reported or available from the Netherlands Cancer Registry, which records clinical data of all newly diagnosed cancer patients in the Netherlands. Additional clinical data (e.g., iodine status at diagnosis, number of I131 treatments, TSH, free T3, free T4, type of surgery, systemic treatment, radiotherapy, dose of radio-active iodine ablation, thyroid hormone substitution type and dose (and possibly switch), current disease status and survival) will be extracted from patients’ medical files.

#### Characteristics of the environment

Food intake (optional) is assessed by asking patients to register all foods and drinks in portion sizes or gram/ml, they have consumed during the day [69] during two weekdays and one weekend-day using the ‘Eetmeter’ from the Dutch ‘Voedingscentrum’ (Netherlands Nutrition Center) at each assessment. The Eetmeter is connected to the Dutch Nutrients Database (NEVO) [70, 71] so the quantity of micro- and macronutrients (i.e., kcal, fat, protein, carbohydrates, vitamins and minerals) will be calculated immediately. Patients receive a link to create an Eetmeter account. When patients have registered their food intake, results will be sent to the secure PROFILES environment [68].

BMI and weight changes are assessed by questions on body height, usual body weight, and current body weight. Also, the questionnaire includes instructions and a tape measure so that patients can assess their waist-, hip-, and calf circumference.

Body composition (optional) is assessed using special weighing scales containing a hand-to-foot BIA system using four pressure contact foot- and four hand pad electrodes (Inbody Dial H20N, Inbody Corporation Europe, Amsterdam, the Netherlands). This indirect method for assessing body composition is based on the electrical conductivity of the different compartments of the human body [72]. BIA measurement can determine the amount of intracellular, extracellular, and total body water. Because fat free mass contains virtually all the body water, BIA methods can be used to estimate non-fat compartments [73]. The InBody DIAL H20N is a two-frequency BIA which sends a current through the body with two frequencies. By using specific formula, fat free mass can be estimated, fat mass is calculated by subtracting fat free mass from body weight. The Inbody Dial is provided 2 weeks at each assessment. Participants are then asked to use the weighing scale each morning immediately after awakening and voiding.

Smoking is assessed by standardized questions on smoking habits (i.e., cigarettes/shag, cigars, pipe tobacco, or e-cigarettes; number of years; number each day). We classify TC survivors in; never, ex, and current smokers.

### Biological mechanisms: inflammation and kynurenine pathway

Blood samples are collected by vena puncture before treatment, 12 and 24 months after diagnosis at the hospitals among all patients using a standard protocol. Participants receive a lab form and a short questionnaire. This questionnaire contains questions concerning medication use, and sickness at the time of blood sampling, as these factors can have an impact on the biological markers of interest. Time of blood donation will be marked on the questionnaire. Directly after blood sampling, the serum blood sample will be allowed to clot at room temperature and is centrifuged. The EDTA blood sample will be centrifuged at room temperature directly after blood sampling. The subtracted plasma, serum, and buffy coat samples will be processed within 2 h of collection and are stored at –80 °C until further analyses. After local processing and temporal storage, the samples will be stored centrally at Biobank Maastricht.

Following, appropriate ELISA and ILLUMINA analyses will take place to determine the biological markers. Blood samples will be stored in the biobank for later analyses of biomarkers.

### Outcomes

Psychosocial outcomes will be assessed with internationally validated questionnaires to assess QOL (EORCT QLQ-C30 [74]), TC-specific symptoms (EORTC QLQ-THY34 [75]), psychological distress (Hospital Anxiety and Depression Scale [76]), fatigue (Multidimensional Fatigue Inventory [77]), sleep (Pittsburgh Sleep Quality Index [78]), personality (optimism, Life orientation test [79]), and comorbidity (Self-administered Comorbidity Questionnaire [80]).

Patients have the option to wear the Fitbit Inspire HR, an activity tracker, which they can keep after the study for personal use as an incentive for participating in the study. They wear the Fitbit for 2 weeks on their non-dominant arm, day, and night. It assesses energy expenditure, metabolic equivalent (MET), exercise intensity, sedentary bouts, activity bouts, steps taken, heart rate, sleep duration, sleep latency, wake after sleep onset, and sleep efficiency.

### Patient feedback

Within WaTCh, we aim to inform patients about TC survivorship for their specific situation: patients can choose to compare their questionnaire scores to their previous scores, to scores of other TC patients, and/or a normative population to see whether their scores are average or not using a traffic light model [81, 82]. Patients with a score in the red part of the chart, so a score that is too high, receive the advice to contact their doctor. Patients are assisted in understanding the graphs. Feedback works well in our other studies; it empowers patients and it is an incentive to participate [83].

### Data analyses

Central to WaTCh is a deep phenotyping approach, meaning that for all patients data will be collected not only at multiple time points but also from different viewpoints (psychological, clinical, environmental, demographic, biological). Jointly analysing these data and making the results insightful requires advanced modelling techniques. To answer the question that belongs to our first objective, we will model physical and psychosocial outcomes over time using multilevel repeated measures models (this is, growth curve models). In addition, data can be explored for subpopulations that have similar profiles over time using latent class variants thereof [84]. A similar type of modelling approach will be used to answer objective two but including covariates. Variants of structural equation models will be used that allow to obtain insight in data consisting of large and multiple collections of variables to answer the question belonging to objective three. Examples of such methods are sparse partial least squares and covariates regression [85, 86].

### Sample size

About 625 Dutch TC patients are diagnosed each year (389 in the included 13 hospitals). Response rates in comparable PROFILES studies are 60% at baseline and 90% at subsequent measurements. Hence, we expect that at least 233 TC survivors respond at baseline, and 210, 189 and 170 respondents respectively at 6, 12 and 24 months after diagnosis which will be sufficient to answer our research questions. In short, to assess the course of physical and psychosocial outcomes over time, 17 participants are necessary to detect a within subjects effect on QOL of d = 0.5, with a power of 0.80, and an alpha of 0,5, given the correlation of 0.6 between the 4 measurement occasions. To assess *who* is at risk for poor outcomes (i.e., QOL), our power analysis indicates that we need at least 152 participants to detect small to medium effects with a power of 0.80. Regarding our third objective, we conducted a power-analysis concerning the hypothesis that depression is associated with C-reactive protein (CRP) over time. For 3 CRP assessments, 2 depression groups and a correlation between the CRP assessments of 0.5, we need 114 participants to discover an interaction effect of d = 0.22 with a power of 0.80.

### Data security/disclosure of original documents

Confidentiality and anonymity of participants will be guaranteed by assigning a study number to each participant. Collected data will all be stored in a secured location (PROFILES registry) for 15 years. PROFILES obtained the Data Seal of Approval in 2016 [87]. The anonymized blood samples will be stored at the Biobank Maastricht.

## Discussion

WaTCh will reveal the course of physical and psychosocial outcomes among TC patients over time and help answer the question *who* is at risk for low physical and psychosocial outcomes, and *why*. This information can be used to inform patients about TC survivorship for their specific situation. Knowing who is at risk for low physical and psychosocial outcomes is also crucial since most patients require lifelong substitution therapy that needs to be adjusted individually. Understanding the driving mechanisms and identifying those at risk for poor outcomes improves personalized treatment, and follow-up. Furthermore, this knowledge can be used to improve screening for physical and psychosocial problems and for developing interventions that could be applied early during the treatment trajectory to optimize outcomes and increase the number of TC survivors that live in good health.

### Trial status

The inclusion of patients started in September 2020. The COVID-19 pandemic delayed patient inclusion.

## Data Availability

After finishing the data collection, the data will be *freely available* for non-commercial scientific research, subject to study question, privacy and confidentiality restrictions, and registration (www.profilesregistry.nl) and the statistical code through www.github.com. This will enable researchers from many different disciplines answer their research questions which will help facilitate dissemination of results and increase the impact of our findings among this vulnerable and understudied population.

## Abbreviations

BIA: Bioelectrical impedance analysis
BMI: Body mass index
CRP: C-reactive protein
DII score: Dietary Inflammatory Index score
MET: Metabolic equivalent
NCR: Netherlands Cancer Registry
NEVO: Dutch Nutrients Database
PROFILES: Patient Reported Outcomes Following Initial treatment and Long term Evaluation of Survivorship
QOL: Quality of life
TC: Thyroid cancer
TSH: Thyroid stimulating hormone
